# Evolutionary and plastic changes in a native annual plant after a historic drought

**DOI:** 10.1002/ece3.6156

**Published:** 2020-04-29

**Authors:** Susan C. Lambrecht, Anjum K. Gujral, Lani J. Renshaw, Lars T. Rosengreen

**Affiliations:** ^1^ Department of Biological Sciences San Jose State University San Jose California

**Keywords:** contemporary evolution, flower size, flowering time, global change, resurrection study, water‐use efficiency

## Abstract

Severe droughts are forecast to increase with global change. Approaches that enable the study of contemporary evolution, such as resurrection studies, are valuable for providing insights into the responses of populations to global change. In this study, we used a resurrection approach to study the evolution of the California native *Leptosiphon bicolor* (true babystars, Polemoniaceae) across populations differing in precipitation in response to the state's recent prolonged drought (2011–2017). In the Mediterranean climate region in which *L. bicolor* grows, this historic drought effectively shortened its growing season. We used seeds collected both before and after this drought from three populations found along a moisture availability gradient to assess contemporary evolution in a common garden greenhouse study. We coupled this with a drought experiment to examine plasticity. We found evolution toward earlier flowering after the historic drought in the wettest of the three populations, while plasticity to experimental drought was observed across all three. We also observed trade‐offs associated with earlier flowering. In the driest population, plants that flowered earlier had lower intrinsic water‐use efficiency than those flowering later, which was an expected pattern. Unexpectedly, earlier flowering plants had larger flowers. Two populations exhibited evolution and plasticity toward smaller flowers with drought. The third exhibited evolution toward larger flowers, but displayed no plasticity. Our results provide valuable insights into differences among native plant populations in response to drought.

## Graphical abstract

In this study, we examined the evolution and plasticity of a native California plant in response to drought. We compared three populations from a moisture availability gradient and found that our wettest population exhibited plasticity in the most traits, while our driest exhibited more evolution, with the exception of flowering time. Our results provide valuable insights into evolution across native plant populations during drought.

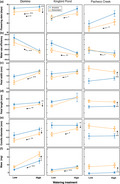

## 1 INTRODUCTION

As the pace of climate change continues to increase, there is more pressing need to study the response of populations to these changes (Parmesan, 2006). Both evolutionary change and plasticity, which is the ability of a genotype to produce different phenotypes in response to environment, are vital to population persistence. Moreover, local adaptation within species may be detected by studying populations along environmental gradients, thus improving predictions of plant responses to climate change.

One expected aspect of global climate change is the altered frequency and intensity of droughts (Swain, Langenbrunner, Neelin, & Hall, 2018). Due to their lack of mobility, plants must be able to endure variations in water availability or suffer declined fitness or even mortality. Therefore, plants have evolved numerous strategies for coping with drought (Ludlow, 1989). Phenological traits, such as flowering time, play a significant role in drought adaptation and are correlated with other key functional traits, such as water‐use efficiency. Some plants escape the effects of drought by developing and reproducing quickly while water is still available (Kenney, McKay, Richards, & Juenger, 2014; Kooyers, 2015; Ludlow, 1989). This strategy may be particularly favored in climates where drought shortens the growing season. However, this rapid growth may require accelerated physiological activity supported by high transpirational water loss, leading to low water‐use efficiency (Geber & Dawson, 1990). Instantaneous water‐use efficiency, or the ratio of carbon gained via photosynthesis to water lost via transpiration, reflects a physiological trade‐off plants face. Intrinsic water‐use efficiency, which is inferred from stable carbon isotope ratios (δ^13^C), is a more consistent, long‐term indicator of water‐use efficiency (Dawson, Mambelli, Plamboeck, Templer, & Tu, 2002). In fact, flowering time is genetically correlated with intrinsic water‐use efficiency, whereby plants that exhibit rapid growth associated with a drought escape strategy will often have low water‐use efficiency (Juenger et al., 2005; Kenney et al., 2014; Lovell et al., 2013; Monroe et al., 2018). Morphological traits are also shaped by drought escape, with rapid growth and low WUE commonly associated with small leaf and flower size, likely due to genetic correlations among development and physiology (Edwards, Ewers, McClung, & Weinig, 2012; Geber & Dawson, 1990).

While escaping drought through rapid growth and physiology is one drought‐coping strategy, another is drought or desiccation avoidance, in which plants grow slowly, flower late, and conserve water through reduced stomatal conductance (Juenger et al., 2005; Kenney et al., 2014; Kooyers, 2015; Ludlow, 1989). While longer growth periods may promote larger leaves and flowers (Geber & Dawson, 1990), small organs may further conserve water by reducing surface area from which water may be lost (Kooyers, 2015; Lambrecht, 2013; Lambrecht & Dawson, 2007). The degree to which an escape versus. avoidance strategy is favored within a population varies with its moisture availability and the time at which drought begins, indicating local adaptation to drought (Edwards et al., 2012; Hamann, Weiss, & Franks, 2018; Heschel & Rignios, 2005; Kooyers, Greenlee, Colicchio, Oh, & Blackman, 2015; Monroe et al., 2018; Sherrard & Maherali, 2006).

Plasticity may further promote survival during drought events. Phenotypical shifts in development rate and intrinsic water‐use efficiency have been exhibited under drought conditions (Gianoli, 2004; Gugger, Kesslering, Stöcklin, & Hamann, 2015; Kenney et al., 2014; Maherali, Caruso, Sherrard, & Latta, 2010). While it is hypothesized that natural selection may favor genotypes that exhibit plasticity in these traits, there is limited supporting evidence (Aspelmeier & Leuschner, 2004; Franks, 2011; Maherali et al., 2010). Therefore, to understand how droughts associated with global climate change affect plant populations, it is essential to study both natural selection and plasticity of drought‐coping traits.

Rapid evolution in response to conditions linked to climate change has been detected for numerous taxa in as few as a couple of generations (reviewed in Parmesan, 2006; Franks, Hamann, & Weis, 2018). One approach used to detect this rapid evolution in plants is the resurrection study, in which seeds collected at one point in time (ancestral) are grown and compared with those collected later (descendant; Etterson et al., 2016; Franks, 2011; Franks et al., 2018; Franks, Sim, & Weis, 2007; Gómez, Méndez‐Vigo, Marcer, Alonso‐Blanco, & Picó, 2018; Hamann et al., 2018; Sultan, Horgan‐Kobelski, Nichols, Riggs, & Waples, 2012; Thomann, Imbert, Engstrand, & Cheptou, 2015; Vigouroux et al., 2011). For plants, this approach is dependent on the collection and storage of seeds over a period in which an environmental change occurs. Using this approach, evolution toward earlier flowering has been identified in crop species (Nevo et al., 2012; Vigouroux et al., 2011) and, in the common, non‐native field mustard (*Brassica rapa* L.) following drought (Franks et al., 2007). However, later studies with the same populations of *B. rapa* observed no plasticity in flowering time associated with experimental drought treatments (Franks, 2011; Hamann et al., 2018).

Between 2012 and 2016, California experienced exceptionally warm and dry weather, resulting in its worst drought in at least 1,200 years (Griffin & Anchukaitis, 2014; NOAA, 2019). As the climate continues to change, forecasts suggest that California may expect more of these extreme drought events (Swain et al., 2018). The recent prolonged drought led to the well‐documented deaths of hundreds of millions of native trees, shrubs, and forbs, including exceptionally long‐lived species like giant sequoia (*Sequoiadendron giganteum* Lindl. [J. Buchholz]) and notably drought‐tolerant chaparral shrubs, like big berry manzanita (*Arctostaphylos glauca* Lindl.; Copeland et al., 2016; Jacobsen & Pratt, 2018; Paz‐Kagan et al., 2017; Prugh et al., 2018; Stephenson et al., 2018; Venturas et al., 2016; Young et al., 2016). Studies have identified several traits, such as deep roots and shifts in stomatal regulation, that have enabled plants to survive and/or recover from the drought (Choat et al., 2018; Jacobsen & Pratt, 2018; Pivovaroff, Cook, & Santiago, 2018; Venturas et al., 2016). This drought also provides the opportunity to study evolution and plasticity of native populations in response to an extreme event.

**Figure 1:**
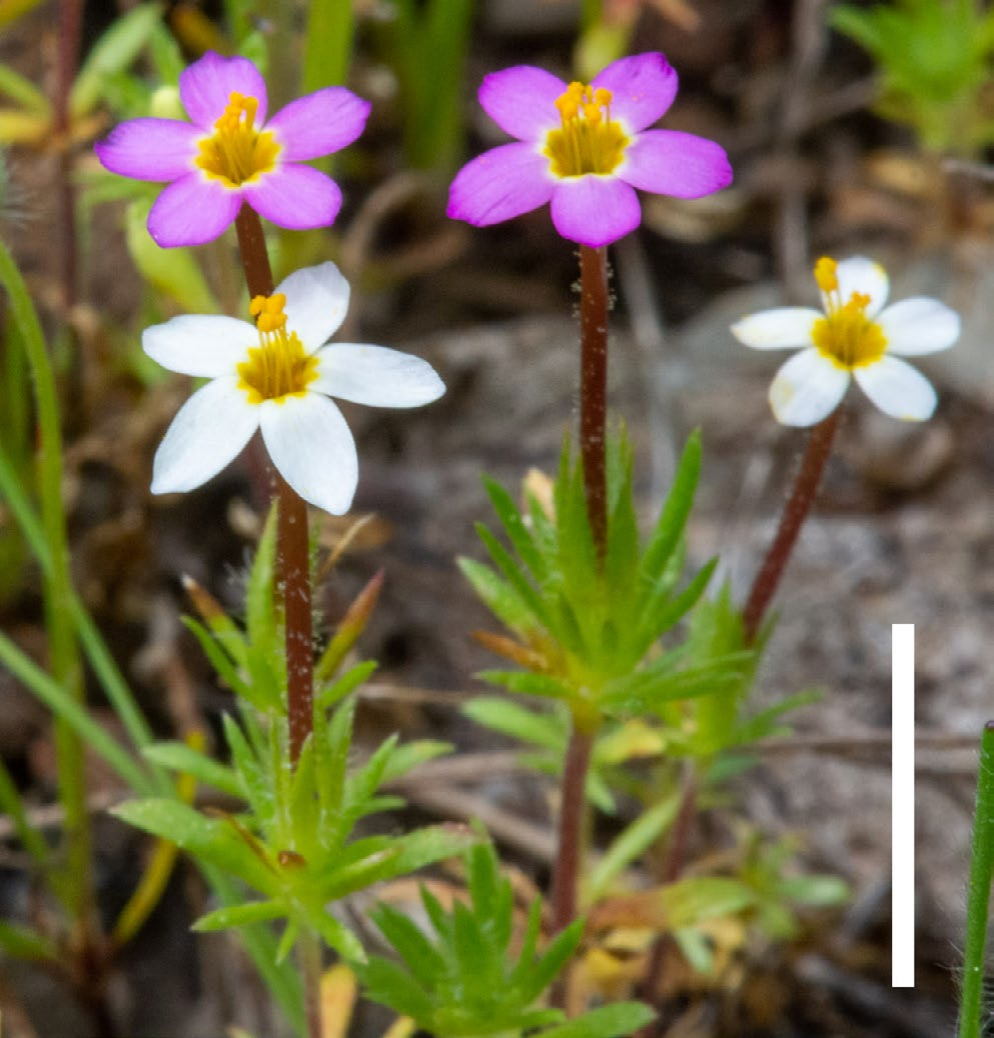
A cluster of *Leptosiphon bicolor* plants, showing the species' flower color polymorphism. The vertical bar = 1 cm

The goal of our study was to test for evidence of evolution and plasticity in a native California annual along a naturally occurring precipitation gradient during the state's recent historic drought. Our study species was *Leptosiphon bicolor* Nutt. (True babystars, Polemoniaceae), which is a highly selfing winter annual found in grasslands and woodlands throughout California and the far western United States and Canada (Figure 1). In these locations, which experience a Mediterranean‐type climate, drought shortens the growing season for winter annuals. Our previous studies with this species have been conducted in populations found along a natural precipitation gradient in the interior coast range of California between 2005 and 2014. In this region, *L. bicolor* germinates with winter rains, flowers in April, and sets seed and senesces by late May. We have found both spatial and temporal variation in plant traits associated with moisture availability, suggesting both heritable and plastic responses (Lambrecht, 2013). Generally, leaves and flowers are smaller in drier populations and years than in moister ones. Fortuitously, during our years of work with this species, we collected seeds from several field populations along the precipitation gradient around the onset of the prolonged drought. We returned to these populations in 2017 to collect postdrought seeds from the same populations. In the current study, we used these seeds to address the following questions: (a) Is there evidence of evolution toward earlier flowering in these populations in response to the drought? (b) Do the plants from the different populations exhibit differences in evolutionary and plastic responses to drought? (c) Is flowering time associated with other traits, such as intrinsic water‐use efficiency or flower size? To answer these questions, we conducted a multiphase resurrection study in a greenhouse on three populations of *L. bicolor* that vary in moisture availability.

## 2 MATERIALS AND METHODS

### 2.1 Study system

**Figure 2:**
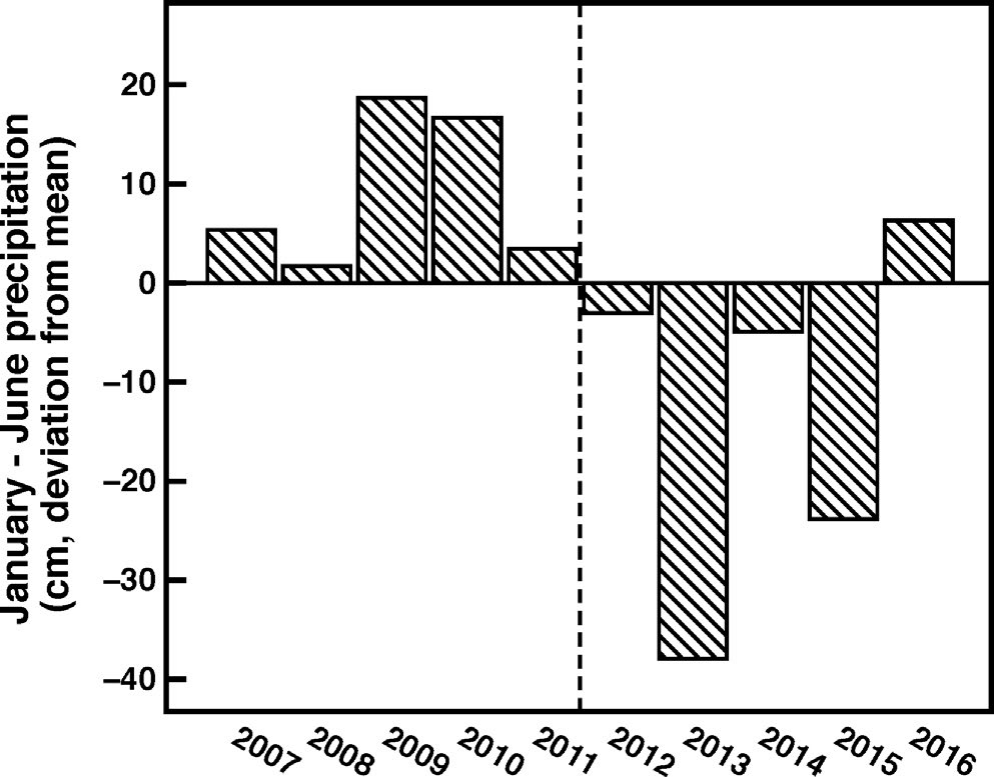
Precipitation at Henry Coe State Park before and during the drought (data from Park, 2019). Values shown are deviations from the average precipitation (44.1 cm) during the growing season of *L. bicolor* recorded between 1975 and 2016 at Coe Park headquarters. The vertical line separates predrought from drought years

The populations of *L. bicolor* used in this study were selected from among those we have previously studied in Henry W. Coe State Park, Morgan Hill, CA (Lambrecht, 2013; L. T. Rosengreen, unpublished data). These populations, located at least 5 km from one another, span a range of moisture availabilities that this species occupies in central California. In order, from wettest to driest, the populations are Domino, Kingbird Pond, and Pacheco Creek. While we were unable to measure precipitation in the populations during the drought, we previously measured precipitation in or adjacent to these locations from 2005 to 2008, which included two wet and two dry years (Lambrecht, 2013). During that time, the 4‐year precipitation average was 71.9 cm for Domino, 38.3 cm for Kingbird Pond, and 26.4 cm for Pacheco Creek (Lambrecht, 2013). Furthermore, the soils at Pacheco Creek are more coarse and sandy than those at Kingbird Pond, causing them to dry more quickly. During the recent prolonged drought, Coe Park experienced overall ~25% reduction of its average precipitation (Figure 2; Henry Coe State Park, 2019). All populations had >1,000 *L. bicolor* individuals. Each plant produces one to several flowers, which have five corolla lobes that are fused into a long corolla tube. The only known pollinator, the long‐tongued fly, has never been observed in these populations, suggesting they are entirely selfing (L. T. Rosengreen, unpublished data). Flowers produce capsules before plants senesce, with each capsule containing ~2–10 seeds. Mature capsules were collected haphazardly from 150 to 200 plants within each population, with seeds separated by maternal line. Collections took place over several days during the peak seed set period of all years; however, this may have led us to miss the earliest and latest seed cohorts. The initial capsule collection year differed across populations, because we did not foresee the drought or this resurrection study at the time of collection (PC: 2011, KBP: 2014, and DOM: 2012). Unfortunately, the different years of collection may have affected the results, since the worst two years of the drought were in 2012 and 2013, prior to the initial collection from KBP. The postdrought seed collection occurred in 2017 for all populations.

### 2.2 Refresher generation

Following the protocol of Franks et al. (2018), we produced a refresher generation of F1 seeds to reduce any possible consequences due to prolonged seed storage or maternal effects. For this generation, we grew field‐collected seeds in a greenhouse at San Jose State University, San Jose, CA. We planted 80 seeds from each population and year, for a total of 480 seeds, using only one seed per maternal line. In November of 2017, one seed was planted in each 18.4 cm deep Cone‐tainer (Stuewe and Sons) filled with moistened Ciardella Mix soil (equal parts redwood compost, horticultural red lava, sand, and peat moss, along with a small amount of fertilizer; Ciardella's Garden Supply). Racks of randomly arranged Cone‐tainers were placed in a seed germinator (Percival Scientific GR‐36L Seed Germinator Chamber) set to a 12 hr day of 10°C and 12 hr night of 5°C. Cone‐tainers were watered daily to keep the seeds moist. After 7 days, the racks were removed from the germinator and placed in a greenhouse away from direct sunlight for 3 days, to allow for acclimation. Then, the racks were removed to an open‐air shade house covered with 50% shade cloth, where plants had access to ambient rainfall and light levels. Additional water was added as needed. Seed germination rates were similar across populations and years (overall mean = 76%; χ^2^ = 0.91, *p* = .34 and χ^2^ = 0.93, *p* = .33, respectively). Potential pollinators are unable to access this greenhouse, so the plants were left to self‐pollinate. Capsules were collected from plants in spring 2018.

### 2.3 Experimental generation

We planted a total of 150 F1 seeds collected from the refresher generation from each population and year combination, for a total of 900 seeds. This total included a minimum of 2 seeds from each of at least 60 F1 maternal lines, with the fully selfed siblings divided between the watering treatments (see below). Our seed germination protocol was the same as that for the refresher generation of the study, with the exception that we planted seeds on three separate dates, due to the capacity of the seed germinator. Seeds from each population and year were divided equally among the planting dates. During this generation, germination rates were ~72%. Following removal from the germinator, the Cone‐tainers were placed in an enclosed greenhouse out of direct sunlight on a bench for 3 days. Then, they were moved under 400 w daylight metal halide lamps (Hortilux Blue) set to a 12 hr day that provided a photosynthetic photon flux density of ~300 µmol m^−2^ s^−1^. Greenhouse temperatures were set to 24°C during the day and 18°C during the night. Relative humidity during the experiment was ~50%. We recorded flowering time as the number of days between germination and flower opening.

Our watering treatment was initiated once plants had acclimated to the greenhouse. For the first two weeks in the greenhouse, all plants were watered daily to saturation. In the third week, we reduced watering to two times per week. Finally, in the fourth week, we ceased all water for the low water treatment, while the well‐watered treatment continued to receive water twice a week. This watering regime was designed to mimic the dry‐down period experienced in field populations during spring, based on our preliminary greenhouse studies. This timeline also enabled us to initiate the drought treatment prior to any flowering.

We observed plants daily to record germination and flowering date. On the date the first flower opened, we measured the width and length of the corolla lobes, as well as the diameter of the corolla face, which are measurements we have previously shown to vary with moisture availability (Lambrecht, 2013). We then collected the aboveground vegetative portion of each plant for stable carbon isotope analysis (δ^13^C) to assess intrinsic WUE. Collected plants were placed in a 60°C drying oven for 72 hr, before being weighed and ground to a fine, uniform sample. Due to the small size of *L. bicolor*, we were only able to analyze 436 of the 499 plants that flowered (87%), since the remainder were too small. A 3.0 ± 1.0 mg subsample was analyzed for δ^13^C on a Delta‐V Isotope Ratio Mass Spectrometer fitted with a Costech Elemental Analyzer at the Facility for Isotope Ratio Mass Spectrometry (FIRMS, University of California). For those plants that died before flowering, we recorded their date of death so we could assess survival across the generations and watering treatments. The experiment was terminated 11 weeks after initial planting. By that time, 63 plants had failed to flower.

### 2.4 Data analyses

To test for evidence of earlier flowering time, we used a Cox proportional hazards model (Fox, 2001). These and all subsequent tests were analyzed using SPSS (v.25, IBM Corp.). Our analyses included generation (ancestral or descendant), population, watering treatment, and block (planting date), as well as all 2‐ and 3‐way interaction terms between generation, population, and treatment. The generation term indicates evolution, while watering treatment indicates plasticity. The interaction of generation × watering treatment estimates whether the scale of plasticity changed after the drought. The Cox model is semiparametric and does not assume a normal distribution, which is essential in testing flowering time, because some plants die before they flower (Fox, 2001). The test generates a Wald chi‐squared test statistic, which is a one‐tailed test with *α* = 0.10. After our initial analysis indicated significant differences between populations (*χ* ^2^ = 9.535, *df* = 2, *p* = .008), we ran post hoc pairwise models to compare populations, using the Sidak‐corrected *α* = 0.034 (Tripathi & Pandey, 2017). Finally, we ran individual analyses of flowering time for each population. Rates of evolutionary change in flowering time were calculated as Haldanes, which calculates unit change per generation, expressed as standard deviations (Gingerich, 2001).

We used mixed models to examine how physiological and morphological traits (intrinsic WUE, flower size, and aboveground vegetative mass) varied among populations over time and with watering treatment using MIXED in SPSS. MIXED uses restricted maximum likelihood methods. Additionally, it only generates type III sums of squares for fixed effects, while random effects contribute only to the covariance structure of the model. Denominator degrees of freedom are calculated using Satterthwaite approximations and are, therefore, not integers. Our initial models included population, generation, and watering treatment as fixed factors, and block as a random factor. For these and the tests described below, *α* = 0.05. We first ran the full model including all 2‐ and 3‐way interactions between population, generation, and treatment. We then ran subsequent models, eliminating nonsignificant interaction terms, one at a time, selecting the model with the lowest value of Akaike's information criteria. When a significant population effect was detected, we used estimated marginal means with a Bonferroni correction to make pairwise comparisons between populations. Finally, intrapopulation models were run to determine the effect of generation and watering treatment within each population.

We used pairwise Pearson correlations to examine the relationship between flowering time and the other measured traits. Correlations were analyzed within each population, generation, and treatment combination.

We assessed survival of plants through the study using the nonparametric chi‐squared test of independence. We compared survival both across the generations and between the watering treatments. We followed these tests with an analysis that compared survival in the low water treatment between generations of each population.

## 3 RESULTS

### 3.1 Evolution in response to a historic drought

**Figure 3:**
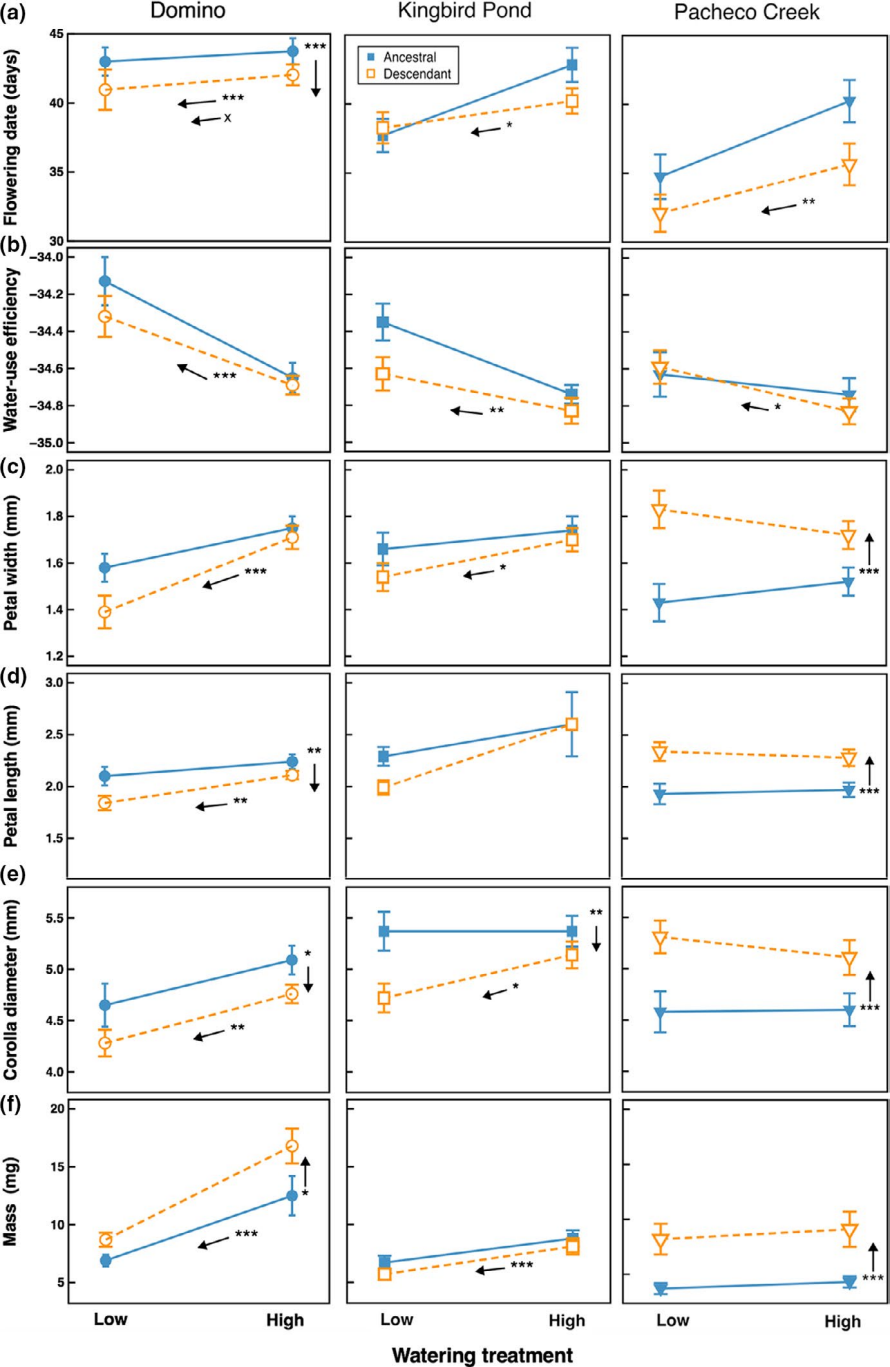
Evolution and plasticity of measured traits. Values shown are mean ± 1 *SE*. Populations are shown, from left to right, in order of wettest to driest. Filled blue symbols are for the ancestral generation, and open orange symbols are for the descendant. Vertical arrows indicate significant effect of generation, revealing evolution. Arrows parallel to the reaction norms indicate significant treatment effects, revealing plasticity in response to watering treatment. The arrow with an X indicates a significant interaction term (*p* < .05) between generation and treatment. See Table 3 for statistics. Bonferroni‐corrected *p < .05, **p < .01, ***p < .001

Evolution toward earlier flowering was detected in one of the three populations. Domino, the wettest population in our study, flowered significantly earlier following the drought, as indicated by a significant generation main effect (Table 1; Figure 3a). In the well‐watered treatment, flowering of Domino plants was 1.7 days earlier in the descendant generation, while in the low water treatment, it was 1.0 days earlier. While the other two populations did not exhibit significant evolution in flowering time, pairwise comparisons indicated there was no statistical difference in response among populations to the drought (pairwise population × generation interactions, all Wald *χ* ^2^ < 4.34, *df* = 1, *p* > .034). Evolutionary rates for each population, calculated in Haldanes, were 0.059 for Domino, 0.057 for Kingbird Pond, and 0.074 for Pacheco Creek. Therefore, although Domino was the only population to demonstrate significant evolution, it did not have the greatest rate of evolution.

**Table 1:** Analysis of flowering time (DTF) evolution and plasticity within each of the populations, listed from wettest to driest

Population	Wald *χ* ^2^			
	Generation	Treatment	G × T	Block
Domino	12.72***	43.02***	5.41*	42.57**
Kingbird Pond	0.01	4.91*	1.18	60.21**
Pacheco Creek	1.26	8.29**	0.14	39.47**

Note

Values shown are chi‐squared statistics from individual Cox regression analyses for each population. For Cox regression analyses, *α* = 0.10 is the level of significance.

* *p* < .05.

** *p* < .01.

*** *p* < .001.

Overall, flowering time differed between Kingbird Pond and Pacheco Creek, which was the driest population in our study, with Kingbird Pond flowering 4 days later than Pacheco Creek (pairwise Wald *χ* ^2^ = 8.36, *df* = 1, *p* = .004). While Domino flowered even later than Kingbird Pond, the difference between it and the other two populations was not significant, using the Sidak‐corrected *α* = 0.034 (pairwise with Kingbird Pond Wald *χ* ^2^ = 0.63, *df* = 1, *p* = .43 and with Pacheco Creek Wald *χ* ^2^ = 3.9, *df* = 1, *p* = .05).

There was more evolution detected in morphological traits than in flowering time (Tables 2 and 3; Figure 3). Domino plants were smaller after the drought, indicated by decreased aboveground biomass, while Pacheco Creek plants were larger (Tables 2 and 3; Figure 3f). In pairwise comparisons between populations, Domino plants had greater biomass than both Kingbird Pond and Pacheco Creek (*p* < .001). While evolution was not detected in floral size traits across the populations (Table 2), our intrapopulation analyses identified differing patterns of evolution within each population (Table 3; Figure 3). All measures of flower size were larger in Pacheco Creek plants following the drought (Table 3; Figure 3c–e). In contrast, Domino showed evolution toward smaller flowers in lobe length and corolla diameter (Table 3; Figure 3d,e), while Kingbird Pond only showed evolution toward smaller corolla diameter (Table 3; Figure 3e). In contrast, evolution of WUE was not detected (Tables 2 and 3; Figure 3b). However, populations did exhibit differences in WUE (Table 2). Domino, which flowered later than the other populations, had higher WUE than both Kingbird Pond (*p* = .006) and Pacheco Creek (*p* = .001).

**Table 2:** Evolution and plasticity of measured traits across populations

Effect	Measured trait				
	Water‐use efficiency (δ^13^C)	Petal width	Petal length	Corolla diameter	Aboveground biomass
Population	7.67**	0.86	2.16	7.67***	23.29***
*df*	428.75	476.32	474	473	**626.45**
Generation	4.47*	2.00	0.001	0.38	16.05***
*df*	430.88	475.93	474	473	626.38
Treatment	33.66***	11.75***	3.02	4.29*	36.05***
*df*	416.07	476.11	474	473	626.07
P × G	NS	10.90***	1.97	13.72***	9.08***
*df*		476.16	474	473	626.33
P × T	NS	4.33*	1.31	2.64	10.61***
*df*		476.01	474	473	626.07
G × T	NS	NS	0.17	NS	NS
*df*			474		
P × G × T	NS	NS	0.23	0.82	NS
*df*			474	473	

Note

Values shown are *F*‐statistics and denominator degrees of freedom. Inclusion of interaction terms was determined by comparing AIC values of alternate models.

*p < .05, **p < .01, ***p < .001, Bold values are significant following the Holm's sequential Bonferroni correction. NS indicates nonsignificant interaction terms that were removed because their inclusion did not improve the model fit.

**Table 3:** Intrapopulation mixed model ANOVAs to test for evolution and plasticity for each trait within each population, listed from wettest to driest

Population	Effect	Measured Trait				
		Water‐use efficiency (δ^13^C)	Petal width	Petal length	Corolla diameter	Aboveground biomass
Domino	Generation	1.21	1.73	7.358**	6.25*	5.38*
	*df*	137	135	135	135	192.43
	Treatment	26.07***	14.64***	9.65**	9.81**	37.13***
	*df*	137	135	135	135	192.1
Kingbird Pond	Generation	4.15*	1.50	0.22	7.85**	2.62
	*df*	157.87	176.50	177	174.26	231.05
	Treatment	12.45**	4.64*	2.10	3.82*	22.77***
	*df*	157.54	178.00	177	175.46	231.01
	G × T	NS	NS	0.21	0.86	NS
	*df*			177	174.59	
Pacheco Creek	Generation	0.19	19.12***	16.74***	13.74***	16.65***
	*df*	132	162.99	163.96	162.61	198.79
	Treatment	4.17*	0.00	0.001	0.06	0.50
	*df*	132	162.80	163.65	162.17	198.22
	G × T	NS	2.32	NS	0.57	0.01
	*df*		161.45		161.23	198.25

Note

Values shown are *F‐*statistics and denominator degrees of freedom. See Figure 3 for mean values.

*p < .05, **p < .01, ***p < .001, Bold values are significant following the Holm's sequential Bonferroni correction**.** NS indicates nonsignificant interaction terms that were removed because their inclusion did not improve the model fit.

### 3.2 Plasticity in response to an experimental drought

All three populations exhibited phenological plasticity by flowering earlier in the low water as compared to the well‐watered treatment. Low water treatment plants flowered about 1 day earlier in Domino (Table 1; Figure 3a), 3.5 days earlier in Kingbird Pond, and 4.4 days earlier in Pacheco Creek. Only Domino exhibited evolution toward increased plasticity in flowering time following the drought (generation × treatment interaction, Wald *χ* ^2^ = 5.41, *df* = 1, *p* = .02). Before the drought, Domino plants flowered 0.75 days earlier in the low water treatment as compared to the well‐watered treatment, but that difference increased to 1.1 days earlier after the drought. Moreover, Kingbird Pond exhibited more plasticity in flowering time in response to the watering treatment than did Domino (pairwise population × treatment interaction, Wald *χ* ^2^ = 9.07, *df* = 1, *p* = .003).

Plasticity was also detected in several other traits. In all populations, plants in the low water treatment had higher WUE than those in the well‐watered treatment, indicating plasticity in WUE (Tables 2 and 3; Figure 3b). While Pacheco Creek showed no plasticity in any morphological traits, Domino plants exhibited plasticity in all traits, producing smaller plants, indicated by reduced biomass, and flowers in the low water treatment. (Table 3; Figure 3). Kingbird Pond plants were smaller in the low water treatment, but floral size traits exhibited no plasticity (Table 3; Figure 3).

### 3.3 Relationship between flowering time and other traits

**Figure 4:**
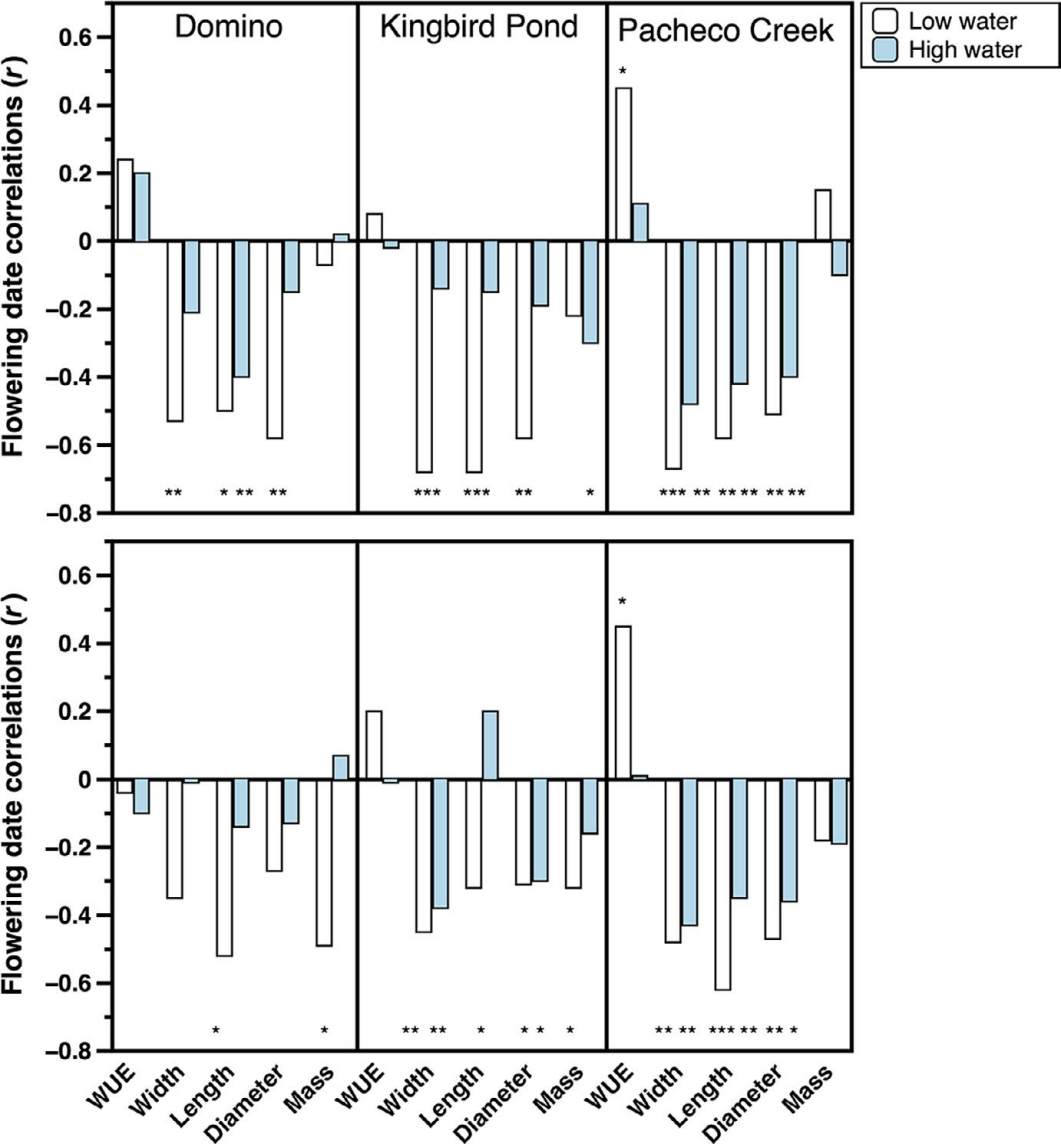
Correlation between flowering time and other measured traits within each of the populations. The upper panels show the ancestral generation, while the lower panels show the descendant. Values shown are Pearson correlations. *p < .05, **p < .01, ***p < .001

Flowering time was more strongly correlated with morphological than physiological traits. Generally, flowers and plants were larger in earlier as compared with later flowering plants (Figure 4). For Domino, these negative correlations were observed more in the low water treatment and in the ancestral generation. For Kingbird Pond, these correlations were more frequent in the low water treatment and the descendant generation. In contrast, Pacheco Creek plants exhibited consistent correlations across generations and water treatments. Furthermore, while it was expected that plants that flowered earlier had lower intrinsic WUE than those that flowered later, this was true only in Pacheco Creek (Figure 4).

### 3.4 Survival

**Figure 5:**
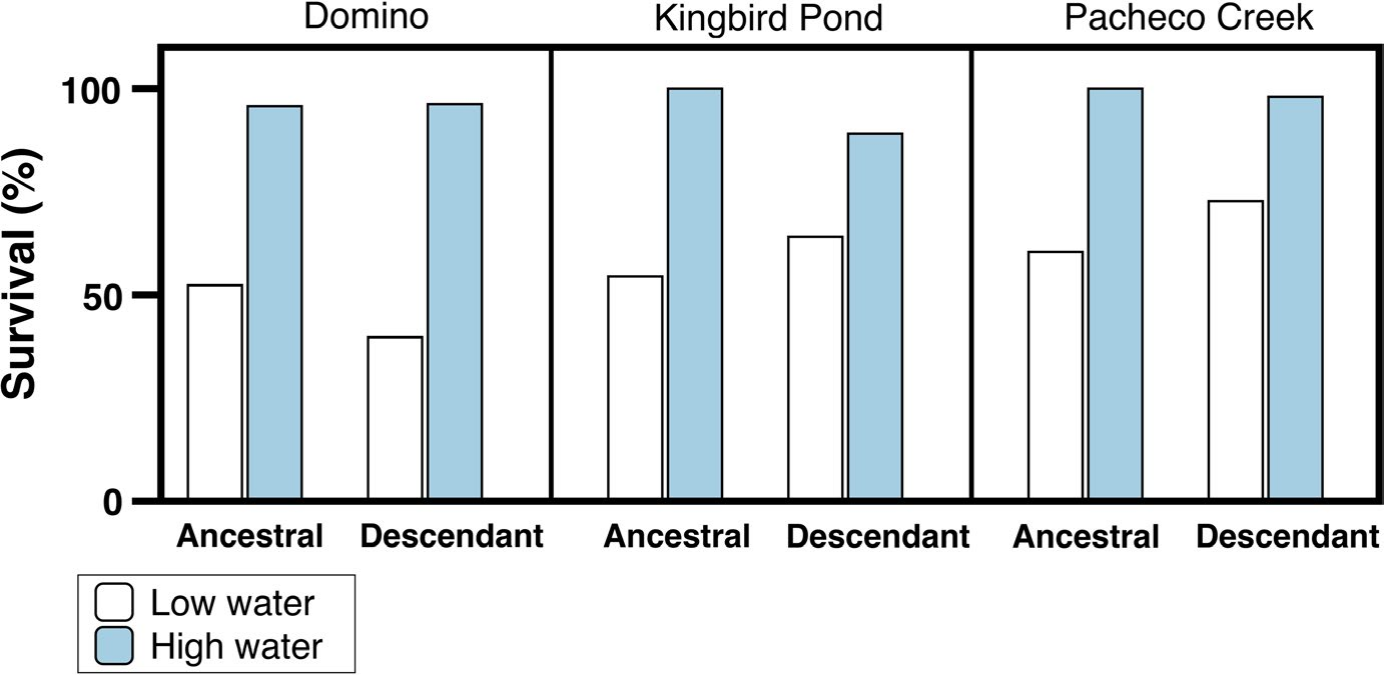
Survival rates across populations and generations. Values are survival (%) for both the ancestral and descendant generations of each population, exposed to the two watering treatments. Survival was lower in the low watering treatment across all populations and generations. None of the descendant generations had improved survival over the ancestral (*χ* ^2^ < 1.54, *df* = 1, *p* > .22)

Survival rates were affected by the watering treatment, but did not change between generations. The low watering treatment reduced survival across all populations (*χ* ^2^ = 145.29, *df* = 1, *n* = 650, *p* < .001). In the low watering treatment, 43% of plants died, while only 3% died in the well‐watered treatment. Survival rates differed among the populations (*χ* ^2^ = 8.81, *df* = 1, *n* = 650, *p* = .01). Domino, the wettest population, suffered the highest mortality (30%), while Pacheco Creek, the driest population, suffered the lowest (17.7%). Across all populations and treatments, both generations had approximately 77% of plants survive (Figure 5; *χ* ^2^ = 0.02, *df* = 1, *n* = 650, *p* = .88). None of the populations had improved survival in the low water treatment in the descendant generation (*χ* ^2^ < 1.85, *df* = 1, *p* > .17).

## 4 DISCUSSION

Our results demonstrate how populations of a native plant species differed in their responses to California's recent historic drought. In the Mediterranean climate region in which *L. bicolor* grows, the recent drought effectively shortened its growing season. However, only the wettest population (Domino) showed evolution toward earlier flowering, although all three populations showed plasticity toward earlier flowering in response to the experimental drought. These results contrast with those of a resurrection study of *Brassica rapa,* which demonstrated evolution, but no plasticity in flowering time in response to natural and experimental drought (Franks, 2011; Franks et al., 2007; Hamann et al., 2018). We also observed trade‐offs associated with earlier flowering, but again, these results differed across populations. Earlier flowering plants in our driest population (Pacheco Creek) had lower intrinsic water‐use efficiency than those flowering later, but only in our drought experiment. Moreover, we detected evolution and plasticity in flower size traits, although the direction and magnitude differed widely across populations, with our driest population showing evolution in the most traits, and our wettest showing the most plasticity. Our results provide valuable insights into the varying drought responses across populations found along a moisture availability gradient.

### 4.1 Evolution in response to a historic drought

In our resurrection study, the population with the greatest moisture availability, Domino, was the only one to exhibit evolution toward earlier flowering. However, in spite of this evolution in flowering time, Domino's plants still flowered later than those of the other two populations. Furthermore, Domino suffered the highest mortality of the populations in our low watering treatment, while Pacheco Creek, our driest population suffered the lowest. In *B. rapa*, earlier flowering was particularly pronounced in the wetter of two populations, presumably because the drier population was already adapted to drought conditions (Franks et al., 2007). Similarly, our drier populations may have already been more well adapted to drought and an earlier end of their growing season than Domino. Additionally, although evolution was significant in only one population, rates of evolution in the populations we studied, calculated in Haldanes, were comparable to those observed for flowering time in *B. rapa* (0.039–0.101; Franks et al., 2007).

Aspects of our study may have limited our ability observe evolution in flowering time. First, seed collection in the field took place over several days during peak seed set for each population. We may, therefore, have missed the earliest and latest seed cohorts, which will have affected our estimates of flowering time. Secondly, our ancestral collection from Kingbird Pond was in 2014, after the worst two years of drought had passed. Therefore, we missed any evolution that may have occurred in the preceding years. This was unavoidable, since we had collected and stored our seeds for other projects and had not anticipated the drought. Finally, due to somewhat low germination rates in our refresher generation, we may have introduced an invisible fraction bias because the phenotypes of the 25% of seeds that did not germinate could not be recorded or used in our estimates of evolution (Weiss, 2018). The bias occurs when seed mortality is nonrandom. However, when we planted sibs in our ancestral generation, we observed no family‐wise pattern in germination, suggesting that failure to germinate was random.

Evolution toward earlier flowering has been demonstrated in a wide array of annual taxa in response to experimental drought treatments, indicating that a drought escape strategy may be favored in annuals so that reproduction and the life cycle may be completed while soil water remains available (Brouillette, Mason, Shirk, & Donovan, 2014; Heschel & Rignios, 2005; Ivey & Carr, 2012; Kenney et al., 2014; Manzaneda et al., 2015; Sherrard & Maherali, 2006). The trend toward earlier flowering is supported by life history theory, which suggests that there are greater fitness costs with flowering later than flowering early (Austen, Rowe, Stinchcombe, & Forrest, 2017; Weis, Wadgymar, Sekor, & Franks, 2014). However, interannual variability in weather, which is predicted to increase with global change, may reverse the selection for early flowering that occurs during dry years and lead to maladaptive responses during intermittent wet years (Hamann et al., 2018). Therefore, rapid evolution in flowering time may not be sufficient to maintain plant fitness under climate change.

While we did not detect evolution in WUE, we did observe that plants that flowered earlier in Pacheco Creek had a lower WUE, which was consistent with a drought escape strategy. Studies have shown flowering time and WUE tend to be more highly correlated under dry conditions (Edwards et al., 2012; Juenger, 2013). Furthermore, Pacheco Creek, which flowered earlier than the other two populations experienced the lowest WUE, while the late‐flowering Domino had the highest. Although a genetic basis for the relationship between WUE and flowering time has been demonstrated in other taxa, selection does not always favor both traits simultaneously, or in the expected direction, and they thus may not be always tightly linked to promote drought escape (Ivey & Carr, 2012; Kooyers et al., 2015; Paccard, Frueleux, & Willi, 2014; Sherrard & Maherali, 2006). Furthermore, the strong genetic correlations between WUE and both flowering time and WUE plasticity identified in other taxa may actually pose genetic constraints on the joint evolution of these traits (Kenney et al., 2014). We could not examine genetic correlations with our study, so are unable to determine whether these may have constrained evolution in WUE in this study.

Evolution was detected in plant and floral size traits. While Domino and Kingbird Pond showed evolution toward smaller flowers and plants, Pacheco Creek showed evolution toward larger flower and plant size. Furthermore, across all populations, earlier flowering plants had greater aboveground biomass and produced larger flowers than later flowering plants, and this pattern was more pronounced in the low watering treatment. These results contrast with those of several studies of annual species that have suggested the rapid development of drought escape leads to smaller flower size in dry environments and may even promote the evolution of a self‐fertilizing mating system (Elle, Gillespie, Guindre‐Parker, & Parachnowitsch, 2010; Emms, Hove, Dudley, Mazer, & Verhoeven, 2018; Ivey & Carr, 2012; Mazer, Dudley, Hove, Emms, & Verhoeven, 2010). However, our results are consistent with those of a resurrection study of cornflower (*Centaurea cyanus*) that documented earlier flowering and larger flowers in plants following several years of warmer springs (Thomann et al., 2015). Furthermore, greater aboveground biomass and flower size in early flowering plants has been documented in numerous annual and perennial species and may reflect the overall condition of the plant. In a recent analysis, 24 of 28 studies surveyed showed a negative correlation between flowering time and plant biomass, suggesting that those that are in better condition (e.g., larger) can flower early and produce larger flowers (Forrest, 2014).

Water loss from flowers may also influence flower size. Work with *L. bicolor* and the closely related *L. androsaceus* has demonstrated that flowers of *Leptosiphon* lose substantial amounts of water, and plants may produce smaller flowers in drier locations or conditions in order to limit that loss (Lambrecht, 2013; Lambrecht, Morrow, & Hussey, 2017). Floral water loss of *Leptosiphon* and other plant species has been shown to affect leaf physiology and can lead to reduced gas exchange, particularly in dry conditions (Dudley, Arroyo, & Fernandez‐Murillo, 2018; Galen, Sherry, & Carroll, 1999; Lambrecht, 2013). In this study, plants from both Domino and Kingbird Pond produced smaller flowers with both the natural and experimental droughts, perhaps because they flowered later after the onset of the terminal drought, when floral water loss would have been more deleterious. In contrast, the earlier flowering plants from Pacheco Creek may have been able to produce larger flowers because they flowered while moisture remained in the soil, so floral water loss was not as costly. The larger mass of descendant Pacheco Creek plants also suggests their rapid development and flower production may have been supported by their overall condition. Studies of floral physiology are lacking, so we have limited knowledge of how floral maintenance costs affect plants, especially as the climate changes.

### 4.2 Plasticity in response to experimental drought

Plasticity in flowering time may be favored as a component of a drought escape strategy to survive where conditions fluctuate from year to year (Austen et al., 2017; Kenney et al., 2014). All populations of *L. bicolor* exhibited significant plasticity by flowering earlier in the low water treatment, although Domino shifted its flowering time the most. Moreover, Domino exhibited evolution toward increased plasticity between the generations. As the wettest of the three populations, drought creates conditions in Domino that are more distinct from those of nondrought years, in comparison with the other populations, which experience dry conditions more consistently. Similarly, increased plasticity in flowering time has been observed for other taxa in moister or more variable environments (Gianoli, 2004; Kenney et al., 2014; Manzaneda et al., 2015). However, increased plasticity is not universally favored. A study of flowering time plasticity in *Arabidopsis thaliana* (thale cress, Brassicaceae) identified selection against plasticity in warmer environments where costs of plasticity were too high to maintain (Stinchcombe, Dorn, & Schmitt, 2004a). Furthermore, long‐term studies with *B. rapa* have found no plasticity in flowering time in response to drought (Franks, 2011; Hamann et al., 2018). Given the different responses across our populations, it is possible that plasticity in flowering time was sufficient to cope with drought in the two drier populations. However, it should be noted that we may have overestimated plasticity in flowering time, due to the invisible fraction bias introduced by the high mortality in the low water treatment. If late‐flowering genotypes died before flowering, it would appear that populations exhibited plasticity toward earlier flowering.

As with flowering time, plasticity in WUE is hypothesized to be favored in variable environments (Kenney et al., 2014). In our study, all populations exhibited plasticity toward increased WUE in the low watering treatment. Therefore, the direction of plasticity of WUE was opposite that expected given the plasticity for earlier flowering time. While such patterns might suggest that the direction of plasticity is maladaptive, similar results have been observed in other taxa (Hamann et al., 2018; Kenney et al., 2014). The time at which the drought treatments were applied may have affected these results, where early droughts have been shown to favor low WUE and early flowering, while later droughts favor increased WUE (Hamann et al., 2018; Heschel & Rignios, 2005). Furthermore, selection analyses reveal that plasticity in WUE, per se, is favored to cope with periodic droughts and enable plants to make use of water while it is available (Kenney et al., 2014). However, few studies have directly tested for the adaptive value of plasticity. Moreover, epigenetics (Zhang, Fischer, Colot, & Bossdorf, 2013) and gene deletions (Stinchcombe, Weinig, et al., 2004b) influence flowering time and its response to environmental conditions, potentially disrupting its relation with other plant traits.

### 4.3 How did populations along a moisture gradient differ in drought response?

Studies of species across broad geographic ranges have revealed differentiation in drought responses, where adaptations favor different combinations of traits associated with both drought escape and drought tolerance across populations, indicating that species do not rely strictly upon one or the other strategy (Bibee, Shishido, Hathaway, & Heschel, 2011; Brouillette et al., 2014; Kooyers et al., 2015; Paccard et al., 2014). Furthermore, differential amounts of plasticity have also been observed across populations in response to drought, with reduced levels of plasticity typically found in more stressful environments (e.g., warmest or driest) or at the margin of a species' range (Gugger et al., 2015; Paccard et al., 2014; Stinchcombe, Weinig, et al., 2004b). In accord with these studies, we observed the least plasticity in the driest of our populations, Pacheco Creek, and the most in the wettest, Domino. Our results suggest that wild populations may have divergent responses to short‐ and long‐term droughts.

In conclusion, our study has demonstrated how rapidly drought can affect responses in a native plant species. We detected both plasticity and evolution in flowering time and flower size in just a few generations, although the magnitude and direction of these responses varied broadly across our three populations. These results provide insight into the variability of evolution across populations, where the role of plasticity versus evolutionary changed varied along the precipitation gradient. Furthermore, extreme drought events and interannual fluctuations in precipitation are predicted to increase as the climate changes. Given that intervening wet years may reverse the selection experienced during drought years (Hamann et al., 2018), there is the need for multiyear studies in a broader array of taxa and across species' ranges in order to understand how populations and species respond to global change.

## CONFLICT OF INTEREST

All authors declare there are no conflicts of interest.

## AUTHOR CONTRIBUTIONS

SCL designed the study and contributed to greenhouse work, data analysis, and manuscript preparation. AKG and LJR contributed to greenhouse and laboratory work and manuscript preparation. LTR contributed to study design, greenhouse work, data analysis, and manuscript preparation.

### Open Research Badges

This article has earned an Open Data Badge for making publicly available the digitally‐shareable data necessary to reproduce the reported results. The data is available at https://doi.org/10.5061/dryad.3tx95x6c3.

## Data Availability

Data are archived on Dryad (https://doi.org/10.5061/dryad.3tx95x6c3).
